# Quantitative profiling of BATF family proteins/JUNB/IRF hetero-trimers using Spec-seq

**DOI:** 10.1186/s12867-018-0106-7

**Published:** 2018-03-27

**Authors:** Yiming K. Chang, Zheng Zuo, Gary D. Stormo

**Affiliations:** Department of Genetics and Center for Genome Sciences and Systems Biology, Washington University School of Medicine, St. Louis, MO USA

**Keywords:** BATF, JUNB, IRF, Transcription factors, Specificity

## Abstract

**Background:**

BATF family transcription factors (BATF, BATF2 and BATF3) form hetero-trimers with JUNB and either IRF4 or IRF8 to regulate cell fate in T cells and dendritic cells in vivo. While each combination of the hetero-trimer has a distinct role, some degree of cross-compensation was observed. The basis for the differential actions of IRF4 and IRF8 with BATF factors and JUNB is still unknown. We propose that the differences in function between these hetero-trimers may be caused by differences in their DNA binding preferences. While all three BATF family transcription factors have similar binding preferences when binding as a hetero-dimer with JUNB, the cooperative binding of IRF4 or IRF8 to the hetero-dimer/DNA complex could change the preferences. We used Spec-seq, which allows for the efficient and accurate determination of relative affinity to a large collection of sequences in parallel, to find differences between cooperative DNA binding of IRF4, IRF8 and BATF family members.

**Results:**

We found that without IRF binding, all three hetero-dimer pairs exhibit nearly the same binding preferences to both expected wildtype binding sites TRE (TGA(C/G)TCA) and CRE (TGACGTCA). IRF4 and IRF8 show the very similar DNA binding preferences when binding with any of the three hetero-dimers. No major change of binding preferences was found in the half-sites between different hetero-trimers. IRF proteins bind with substantially lower affinity with either a single nucleotide spacer between IRF and BATF binding site or with an alternative mode of binding in the opposite orientation. In addition, the preference to CRE binding site was reduced with either IRF binding in all BATF–JUNB combinations.

**Conclusions:**

The specificities of BATF, BATF2 and BATF3 are all very similar as are their interactions with IRF4 and IRF8. IRF proteins binding adjacent to BATF sites increases affinity substantially compared to sequences with spacings between the sites, indicating cooperative binding through protein–protein interactions. The preference for the type of BATF binding site, TRE or CRE, is also altered when IRF proteins bind. These in vitro preferences aid in the understanding of in vivo binding activities.

**Electronic supplementary material:**

The online version of this article (10.1186/s12867-018-0106-7) contains supplementary material, which is available to authorized users.

## Background

The signature characteristic of basic leucine zipper (bZIP) transcription factors is the alpha-helical bZIP domain that contains both a DNA binding region and a leucine zipper motif. The leucine zipper motif allows bZIP transcription factors to form either hetero- or homo-DNA binding dimers [1]. One of the most well-known examples of hetero dimerizing bZIP transcription factors is the FOS–JUN dimer which is also known as activator protein 1 (AP-1). AP-1 family proteins are known to be able to regulate gene expression either on their own, or with a partner via closely spaced DNA-binding sites [2, 3]. Basic leucine zipper transcription factor ATF-like (BATF) family transcription factors (BATF, BATF2, and BATF3) belong to the family of bZIP transcription factors and are considered as AP-1 transcription factors due to their DNA binding preferences. BATF family proteins form hetero-dimers with JUN family proteins and can recognize the 7-long TPA response elements (TRE: TGA(C/G)TCA) or the 8-long cyclic AMP response element (CRE: TGACGTCA) [4–6]. The bZIP domain of all three BATF family members are highly conserved. None of the BATF transcription factor have a transcriptional activation domain, and are considered to act as inhibitors of AP-1 activity [7]. BATF and BATF3 are relatively small compared to other bZIP transcriptional factors (125 and 118 amino acids, respectively) and contain no additional domains other than bZIP. BATF2 has an extra carboxy-terminal domain of unknown function.

mRNA expression analysis showed that BATF and BATF3 were highly expressed in lymphocytes while BATF2 is mostly expressed in macrophages [8]. While sometimes expressed in the same cell types, each BATF family member has specific functions. For example, BATF is found to control TH17 differentiation [9] and BATF3 is required for the development of CD8a classical dendritic cells (cDC) [10]. Interestingly, BATF and BATF3 can cross-compensate in vivo in T cells and dendritic cells, but BATF2 can only compensate for BATF3 in dendritic cells [11]. The mechanism for how the family members compensate for each other is not clear.

Interferon regulatory factors (IRFs) family transcription factors have diverse roles in regulating the immune system. IRFs have a conserved DNA binding domain (DBD) known to bind to the interferon-stimulated response element (ISRE) by itself [12, 13]. While the mammalian IRF family comprises nine members from IRF1 to IRF9, only IRF4 and IRF8 are known to cooperatively function with BATF family transcription factors. Structurally, IRF4 and IRF8 contain an IRF-association domain (IAD) C-terminal to the DBD. When binding cooperatively with BATF, the IAD is proposed to interact with the leucine zipper region on the BATF and the DBD binds to “GAAA” motif either 0 or 4 base pairs away from the TRE in opposite orientations [11, 14, 15].

The basis for the differential actions of IRF4 and IRF8 with BATF factors is still under investigation. One potential explanation could be the subtle differences in cooperative DNA binding between BATF factors and IRFs. Iwata et al. found that a “T” preference 8 base pairs 5′ to the TRE can affect the strength of T cell antigen receptor signal [16]. We propose that the differences in function between these hetero-trimers is caused by differences in their DNA binding preference. We used Spec-seq, which allows for the efficient and accurate determination of relative affinity to a large collection of sequences in parallel [17–21], to find differences between cooperative DNA binding of IRF4, IRF8 and BATF family members.

Spec-seq is based on the principle that the relative binding affinities of a collection of DNA sequences can be measured by separating the bound and unbound fractions of DNA and determining the ratios of each sequence in the two fractions (see “Methods”). We have used this principle to measure binding specificity many times previously, but with methods that were low-throughput, allowing the measurement of relative affinity to only a few sequences per assay [22–27]. With the development of new sequencing technologies, Spec-seq allows that principle to be applied to measure the relative binding affinities of hundreds to thousands of sequences per assay [17–20, 28]. We have recently demonstrated that it can be easily extended to measure the effects of modified bases on binding affinity, and also showed its high accuracy by comparison with a two-color competitive fluorescence anisotropy method [21]. Spec-seq can also be readily adapted to measuring the cooperativity of binding between two proteins to the same DNA sequence, in a method we call Coop-seq [17, 29, 30]. In this paper Spec-seq is applied for the first time to the study of hetero-trimeric protein-DNA complexes.

## Results

### Spec-seq of BATF/BATF2/BATF3 with JUNB

**Fig. 1:**
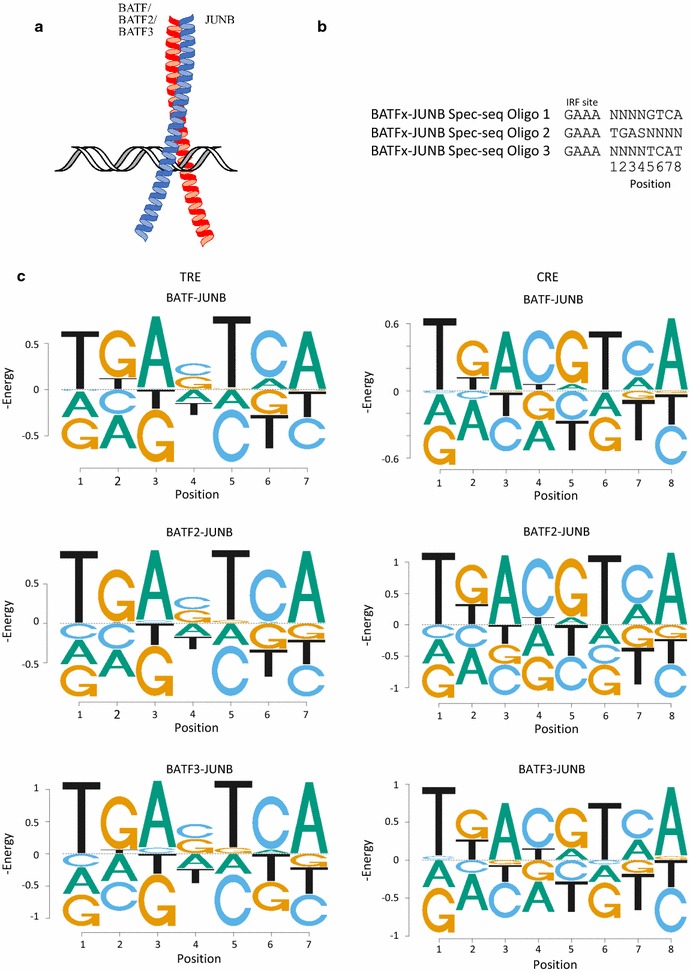
Spec-seq of BATF/BATF2/BATF3 with JUNB. **a** BATFx dimerizes with JUNB to bind to DNA. **b** Oligos used to generated the library used in the Spec-seq experiment. Only the binding sites are shown. Each of these sequence in the library is flanked with sequences for amplification purposes as described in “Methods”. **c** Energy logos for BATFx–JUNB heterodimers for both TRE and CRE binding sites. Since these binding sites have no directional preferences, these logos are generated as symmetrical. Single variants from the consensus BATFx–JUNB binding site of GAAA were used to generate these logos. The Y-axis is negative energy in kT units, so the preferred sequence is on the top. Energy PWMs are in Additional file 3

We used full length human BATF and BATF3 and the bZIP domain of BATF2 (142aa). BATFs were heterodimerized with JUNB prior to protein purification. Each BATFx–JUNB hetero-dimer was incubated with the Spec-seq library to induce DNA-BATFx–JUNB binding (Fig. 1a). The binding reactions were loaded onto native polyacrylamide gels for electrophoretic mobility shift assay (EMSA) (Additional file 1). The separated bound and unbound bands on the gel were extracted separately for DNA, then sequenced by Illumina sequencing. The read-counts of each oligo were used for Spec-seq calculation of relative binding affinity [17, 18] (see “Methods”). The DNA library used here contained three oligos (Fig. 1b). Oligos 1 and 2 can be bound in either the CRE (TGACGTCA) or TRE (TGA(C/G)TCA) mode, whereas oligo 3 can only be bound in the TRE mode. For the TRE sequences there is a single randomized flanking position which we find does not contribute to specificity, consistent with our previous results [21]. Spec-seq calculations generated relative binding energies for each of the oligos used in the library (Additional file 2). Energy logos were drawn by using only the single variant mutants from either CRE or TRE reference (energy PWMs are included in Additional file 3). All three BATF–JUNB combinations have a similar preference of binding to the TRE and CRE sites (Fig. 1c). BATF binds the TRE and CRE sites with approximately equal affinity, while BATF2 and BATF3 have a preference for CRE of 0.3 – 0.4 kT (Additional file 4). Our result agrees with Rodriguez-Martinez et al. [31], who also used heterodimers of all three BATFs bZIP domain with JUNB. The BATF2–JUNB combination is especially of note because previous reports of the full length BATF2 and JUNB combination failed to bind to TRE [32, 33].

### IRF4 and IRF8 spec-seq with BATF/BATF2/BATF3 and JUNB

**Fig. 2:**
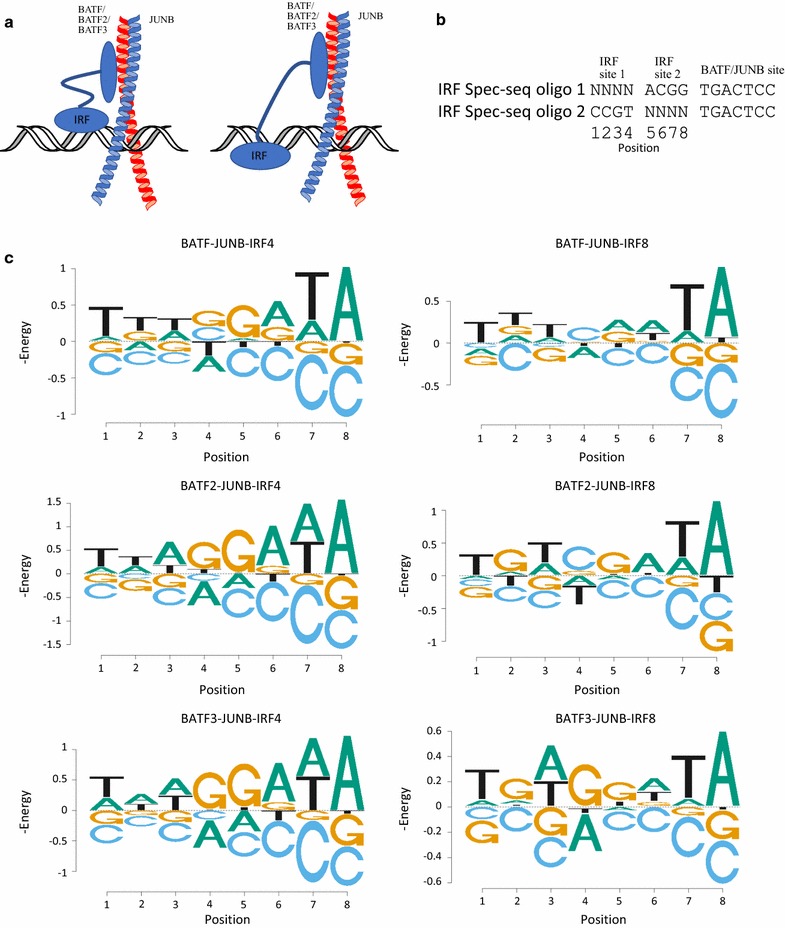
IRF4 and IRF8 spec-seq with BATF/BATF2/BATF3 and JUNB. **a** Two modes of potential BATFx–JUNB–IRFx hetero-trimer binding to DNA [14]. The IRFx can bind either 0 or 4 nucleotides away from the BATFx–JUNB binding site. **b** Oligos used to generated the library used in the Spec-seq experiment. Each oligo contains two potential IRF binding locations, either 0 or 4 nucleotides from the BATFx–JUNB binding site. The IRF site intended for binding test is randomized to NNNN while the IRF site not intended for IRF binding was mutated to ACGG, a sequence not preferred by either IRF. The BATFx–JUNB site is mutated to have a “C” instead of an “A” on the 7th position to facilitate BATFx–JUNB binding in only one direction. **c** Energy logos for BATFx–JUNB–IRFx hetero-trimer binding. Logos for two IRFx sites were generated separately and combined in a single logo. Single variants from the consensus IRFx binding site of GAAA were used to generate these logos. The Y-axis is negative energy (kT units) so the preferred sequence is on the top

IRF4 and IRF8 have low affinity to DNA on their own. When subjected to Selex experiments, a “GAAA” rich motif known as ISRE can be found [13]. However, that cannot reflect the realistic binding situation in vivo. Glasmacher et al. [14] reported that IRF4 Chip-seq experiment from TH17 cells yields motifs with a “GAAA” either 0 or 4 bases away from the AP-1 site. The 0-spacer “GAAA” and 4-spacer “TTTC” binding site suggests that the IRF could have two modes of DNA binding with different spacers and orientations (Fig. 2a). We designed our oligo library to measure the relative DNA binding affinity of IRFs under the presence of BATF–JUNB. The library contains oligos with randomized potential IRF sites. To allow only one potential IRF binding per protein-DNA complex, we changed the non-randomized positions to sequences that were determined in prior Spec-seq experiments to be a non-preferred sequence (ACGG). Since AP-1 sites are palindromic, we mutated the distal half of AP-1 binding site to a lower preference one (TCC instead of TCA) because IRF was shown to prefer binding to the more conserved side of the AP-1 site [14] (Fig. 2b). As in the BATF–JUNB Spec-seq experiments, IRF–BATF–JUNB and the DNA library were incubated and then run on native polyacrylamide gels for EMSA experiments (Additional file 1). Bound and unbound bands in the EMSA experiments were extracted for DNA and sequenced through Illumina sequencing to produce read counts for Spec-seq calculation. Energy logos were drawn by using only the single variant mutants from either “TTTC” or “GAAA” references for 4 and 0 spacers respectively, then merged together (energy PWMs in Additional file 3). Overall, the 0 spacer sites (position 5–8) for both IRF4 and IRF8 have higher specificity than the 4 spacer sites (position 1–4) (Fig. 2c). The two bases closest to the AP-1 site contribute the most to those preferences. Both IRF4 and IRF8 0-spacer half site show up as “GA(T/A)A.” IRF4 prefers A and T equally on the third position of the IRF site while IRF8 prefers a T at the third position. The binding affinity is much higher with the 0 spacer sites than with the 4 spacers, and the magnitude of the difference depends on both the IRF protein and the BATF dimer (Additional file 5). For IRF4, BATF and BATF3 both have about 1.6 kT higher affinity for the 0 spacer, whereas for BATF2 the effect is about 2.4 kT. For IRF8, BATF and BATF3 prefer the 0 spacer site by about 0.6 kT, whereas BATF2 prefers the 0 spacer site by about 1.1 kT (Additional file 5).

### Change in BATF/BATF2/BATF3 and JUNB specificity with IRF4 and IRF8 binding

**Fig. 3:**
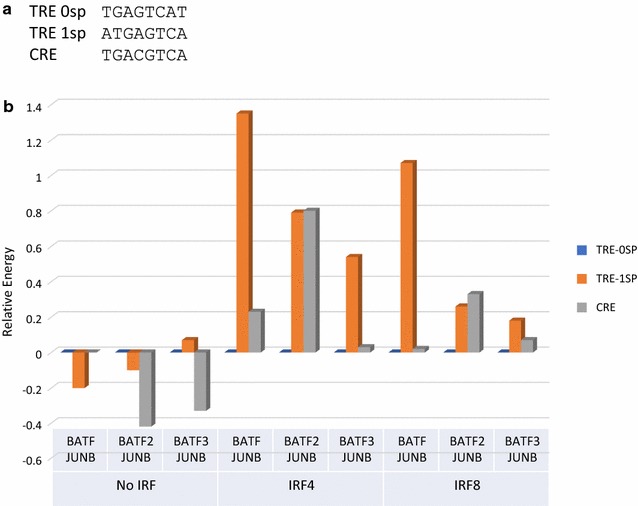
Change in BATF/BATF2/BATF3 and JUNB specificity with IRF4 and IRF8 binding. **a** Targets sequences used for binding energy comparison. **b** Relative binding energy for each protein combination and target sequence listed. To compare across different experiments, each relative energy measurement was normalized by setting the energy of TGAGTCAT (TRE-0sp) to 0. The higher energy value represents lower affinity

To find out if BATF/BATF2/BATF3–JUNB DNA binding preferences would be affected by IRF4/IRF8 binding, we combined BATF/BATF2/BATF3–JUNB hetero-dimers with either IRF4, IRF8 to form DNA binding hetero-trimers and measured their relative DNA binding affinities using Spec-seq with the DNA libraries shown in Fig. 1b. We focused on comparing the binding energy of both TRE with no spacer (TRE-0sp), CRE and the artificial condition of IRFx–BATFx–JUNB binding to TRE with 1 nucleotide spacer between the IRF site and TRE (TRE-1sp). Oligos with hamming distance of 1 from TRE-0SP, TRE-1SP, and CRE (Additional file 6A) were used to generate energy logos of half-site binding preferences for BATF/BATF2/BATF3–JUNB hetero-dimers and BATF/BATF2/BATF3–JUNB–IRF4/IRF8 hetero-trimers (energy PWMs in Additional file 3). No major change of binding preferences was found in the half-sites (Additional file 6B). However, there is large change in binding energy when IRFx is binding with BATFx–JUNB for different consensus binding sites (TRE-0sp, TRE-1sp, and CRE) (Additional file 4) (Fig. 3a). We first normalized all energy measurements by setting TGAGTCAT (TRE-0sp) measurements in each experiment to 0. The binding energies of TGAGTCAT (TRE-0sp), ATGAGTCA (TRE-1sp) and CRE for each protein combination are graphed (Fig. 3b). The higher energy value represents lower binding affinity. As described above, with no IRF binding, BATF2 and BATF3 show a small preference for CRE versus TRE-0sp (with no IRF, TRE-0sp and TRE-1sp are equivalent sites; the measured differences are within the experimental uncertainly of about 0.2 kT). However, when IRF4 is involved in binding with the hetero-dimers, all three hetero-trimers bound to TRE-0sp 0.6 and 1.4 kT better than to TRE-1sp. This result suggests that the BATF/BATF2/BATF3–JUNB–IRF4 trimer formation is sensitive to the amount of spacer between IRF and BATF and that binding in an adjacent position shows cooperativity. The results are similar, although somewhat lower in magnitude, for IRF8. In contrast, the CRE sites have nearly equal affinity to the TRE 0 spacing sites for both IRF4 and IRF8 with both BATF and BATF3. But CRE sites have lower affinity, equivalent to TRE 1 spacer sites, for both IRF4 and IRF8 with BATF2.

## Discussion

We have found that quantitative specificities of BATF, BATF2 and BATF3 are all very similar over a large collection of binding sites. The main difference being that BATF2 and BATF3 have a slight preference for 8-long CRE sites over 7-long TRE sites that is not observed for BATF. IRF4 and IRF8 have very similar specificities in combination with any of the BATF proteins. In every case there is a preference for IRF sites that are immediately adjacent, 0 spacer sites, to those that have a single base in between, which strongly suggests cooperative binding through protein–protein interactions [29]. The preference for the 0 spacer sites over the 4 spacer sites, with the IRF site in the opposite orientation, is even stronger. The fact that such combinations are observed in in vivo binding sites [14] suggests that there are other, currently unknown, factors contributing to the complex formation in vivo. Although the specificities of the BATF proteins are very similar, as are those of the IRF proteins, there are some significant differences in the interaction energies that may account for differential binding in vivo.

## Conclusions

BATF, BATF2 and BATF3 each can form dimers with JUNB and bind DNA with very similar specificities. Each dimer can also interact with IRF4 and IRF8 to form hetero-trimeric protein complexes that bind to DNA with similar, but somewhat distinct quantitative preferences, especially regarding the spacings between the monomeric sites. Spec-seq is an effective method to measure the relative affinities to hundreds of alternative binding sites in parallel.

## Methods

### Protein expression and purification

#### BATF/BATF2/BATF3–JUNB heterodimers

Full length human BATF, BATF3 and a truncated version of BATF2 (aa 1–142) were cloned into a pUC19 based plasmid with T7 promoter and T7 terminator. Only the N-terminal bZIP domain of BATF2 was used to make it equivalent to BATF and BATF3 and because earlier work had shown that the full length BATF2 did not bind TRE sequences with JUNB [32, 33]. Each protein construct contains a N-terminal mCherry followed by a cleavage site for Tobacco Etch Virus nuclear-inclusion-a endopeptidase (TEV protease) and finally the actual protein of interest. In addition, a truncated version of human JUNB (aa 148–347) with C-terminal 6-histidine (6His) tag were cloned into a pBR322 based plasmid with T7 promoter, T7 terminator, Kanamycin resistance and no rop gene. Each BATF plasmid was co-transformed with the JUNB plasmid into SHuffle T7 Express Competent E. coli (NEB) and grown in Luria broth LURIA BROTH (Sigma). Protein expression was induced by adding 0.4 mM isopropyl-B-thiogalactoside (IPTG) for 16 h at 25 °C. The proteins were purified using Ni–NTA agarose (Qiagen) following manufacturer’s instructions, mCherry-colored flow through were collected. The mCherry on BATF proteins serves as an indicator for mCherry-BATF existence. Since the BATF proteins contain only mCherry and no affinity tags, all 6His purified proteins were hetero-dimerized BATF–JUNB. The mCherry on BATF proteins were cleaved off by using ProTEV Plus (Promega) following manufacturer’s instructions.

#### IRF4/IRF8

Full length human IRF4 and mouse IRF8 were cloned into a pUC19 based plasmid with T7 promoter and T7 terminator containing N-terminal strep-tag followed by cleavage site for thrombin protease as described (39). The construct was transformed into Escherichia coli BL21(DE3) and grown in LURIA BROTH (Sigma). Protein expression was induced by adding 0.4 mM isopropyl-B-thiogalactoside (IPTG) for 3 h at 30 °C. The proteins were purified using Strep-Tactin Superflow (IBA Life Sciences) following the manufacturer’s instructions. The strep-tag was cleaved off by thrombin protease digestion for 8 h at room temperature.

### Library design and preparation

The BATF–JUNB Spec-seq library was designed by flanking the degenerate sequences of interest (those in Fig. 1b) with 5′ flanking sequence of GATAGTCTCATTTTCACCCCGT and 3′ flanking sequence of TTGTTCCATTACAGTATCTGT for downstream processing. The IRF Spec-seq library was designed by flanking the degenerate sequences of interest (those in Fig. 2b) with 5′ flanking sequence of GAGTCGTCTCGTCAGCACTA and 3′ flanking sequence of CCGTAGAGCACTCAGGTC for downstream processing. Libraries were procured by ordering single stranded DNA oligos from IDT. To make double-stranded DNA (dsDNA) libraries, 100 pmol single-strand degenerate template sequences were mixed with an equal amount of appropriate reverse complement primer (ACAGATACTGTAATGGAAC or GACCTGAGTGCTCTACGG). In the presence of Taq Polymerase (Lambda Biotech), brief 10-s denaturing followed by 10 min of 55 °C annealing/extension is sufficient to make dsDNA libraries. Because any unextended single-stranded DNA (ssDNA) could contaminate the unbound band, the reaction mix was digested by 1 ml NEB Exo I exo-nuclease (New England Biolabs) for 30 min. All final dsDNA products were purified by PCR purification columns (QIAGEN) and eluted in MilliQ water (Millipore).

### Spec-seq experiments

All binding reactions were done in a 10 µl reaction volume using 100 nM BATF proteins-JUNB heterodimers, 150 nM IRF proteins if needed, 1μM of dsDNA library in 1× NEB Cutsmart buffer (50 mM Potassium Acetate; 20 mM Tris–acetate; 10 mM Magnesium Acetate; 100 μg/ml BSA, pH 7.9 @25 °C) supplemented with 10% glycerol and were incubated for 30 min on ice. Electrophoresis mobility shift assays (EMSA) were done using native 9% PAGE prepared as Tris/Glycine (25 mM Tris pH 8.3; 192 mM glycine) mini-gels (Bio-Rad). These gels were first pre-run using 1× Tris/Glycine buffer at 200 V for 30 min, then samples were loaded and gels were run for an additional 40 min at 200 V at 4 °C. After EMSA, the gels were stained with ethidium bromide and visualized using Bio-Rad gel imager. Each band detected in the EMSA were excised with a disposable sterile toothpick and the DNA in the gel extracted by incubating for 30 min at 50 °C in 50μl acrylamide gel extraction buffer [500 mM Ammonium acetate; 10 mM magnesium acetate; 1 mM EDTA; 0.1% sodium dodecyl sulfate (SDS)]. Samples in the extraction buffer were purified with QIAquick Nucleotide Removal Kit (Qiagen) following the manufacturer’s instructions and recovered using MilliQ water (Millipore). Each fraction of DNA was barcoded and amplified using HotStart PCR Master Mix (Lambda Biotech). DNA was denatured at 94 °C for 30 s, annealed at 55 °C for 30 s and extend at 72 °C for 45 s per round for 12–20 rounds with modified Indexed-Illumina primers (PE1-Genetics1/2, PE2.0) (Additional file 7). The PCR product was then purified again using QIAquick Nucleotide Removal Kit. Multiple samples were pooled and sequenced and analyzed as previously described [18].

Analysis of Spec-seq data to determine relative binding energies for a collection of sequences is as previously described [17, 18]. Briefly, the affinity (association constant) of a TF to any sequence, *S*_*i*_, can be determined by measuring the concentrations of the unbound TF, the unbound *S*_*i*_ and the TF-*S*_*i*_ complex ([TF], [*S*_*i*_], [TF-*S*_*i*_], respectively)

$$K_{A} \left( {TF,\;S_{i} } \right) = \frac{{\left[ {TF \cdot S_{i} } \right]}}{{\left[ {TF} \right]\left[ {S_{i} } \right]}}.$$

To obtain the relative affinity of the TF to a collection of sequences, *S*_1_…*S*_*n*_, (which for convenience we label *K*_1_…*K*_*n*_) requires only measuring the distribution of those sequences in the bound and unbound fractions and the none of the concentrations, including that of the free protein, are needed:

$$K_{1} :K_{2} : \cdots :K_{n} = \frac{{P\left( {S_{1} |B} \right)}}{{P\left( {S_{1} |U} \right)}}:\frac{{P\left( {S_{2} |B} \right)}}{{P\left( {S_{2} |U} \right)}}: \cdots :\frac{{P\left( {S_{n} |B} \right)}}{{P\left( {S_{n} |U} \right)}}$$

where *P*(*S*_*x*_|*B*) and *P*(*S*_*x*_|*U*) refer to the probabilities of sequence *S*_*x*_ within the bound and unbound fractions, respectively.

## Additional files

**Additional file 1.** EMSA gel of protein-DNA complexes. An example of an electrophoretic mobility shift assay (EMSA) gel with proteins BATF3, JUNB and IRF8 and randomized DNA libraries BATFx–JUNB Spec-seq Oligo 1–3 (Fig. 1). First lane shows band for unbound DNA. Second lane includes band for BATF3/JUNB complex with DNA (bound band). Fourth lane includes BATF3/JUNB/IRF8 and shows a more diffuse band higher in the gel than the band in lane 2. The gel picture on the right is taken after cutting out the bands and shows the extent of each band that is cut out for DNA extraction and sequencing.

**Additional file 2.** Spec-seq results for BATFx-JUNB. Spec-seq results for BATF-JUNB (sheet1), BATF2-JUNB (sheet2), and BATF3-JUNB (sheet3). Each sheet contains a CRE part and TRE part. Only the single variants from the wild type target (TGACGTCA for CRE and TGAC/GTCA for TRE) were included in the tables. The number of counts in the bound and unbound bands are provided. The ration of bound/unbound are proportional to the relative binding affinities. Energy = −ln(ratio); energy units are kT (k = Boltzmann constant, T in degrees Kelvin).

**Additional file 3.** Energy PWMs from each experiment. For each binding reaction an energy PWM is determined from the consensus sequence and the energy differences for all single variants of the consensus. Each PWM is labeled with the figure of the Logo based on that PWM.

**Additional file 4.** Normalized binding energy for BATFx-JUNB-IRFx for TRE/CRE sites. Normalized binding energy for BATFx-JUNB-IRFx for binding sites TRE-0sp, TRE-1sp and CRE are shown. Energy value were taken from TGAGTCAT for TRE-0SP, ATGAGTCA for TRE-1sp, and TGACGTCA for CRE and normalized by setting the energy value of TGAGTCAT of each TF combination to 0 (Fig. 3).

**Additional file 5.** Normalized binding energy for BATFx-JUNB-IRFx for IRF sites. Normalized binding energy for BATFx-JUNB-IRFx for IRF binding sites are shown. Energy value were taken from averaging the energy of all oligos single variant to the wild type binding site (GAAA for 0sp and TTTC for 4sp), then normalized by setting the 0-sp for each TF combination to 0.

**Additional file 6.** Half site analysis for BATFx-JUNB-IRFx. (A) Single variants from half sites of these oligos in the library were used to generate energy logos. Bolded positions represent the half sites generated in B. (B) Energy logos from Spec-seq results of BATFx-JUNB-IRFx. The Y-axis is negative energy so the preferred sequence is on the top.

**Additional file 7.** Oligos used. Oligos used for PCR and sequencing.
