# Supplementary figures and images for: Quantitative profiling of BATF family proteins/JUNB/IRF hetero-trimers using Spec-seq
Source: BMC Mol Biol. 2018 Mar 27;19:5. doi: 10.1186/s12867-018-0106-7 (PMC5869772; doi:10.1186/s12867-018-0106-7)

Before

|            |   |   |   |   |
|------------|---|---|---|---|
| DNA        | + | + | - | + |
| BATF3/JUNB | - | + | - | + |
| IRF8       | - | - | - | + |

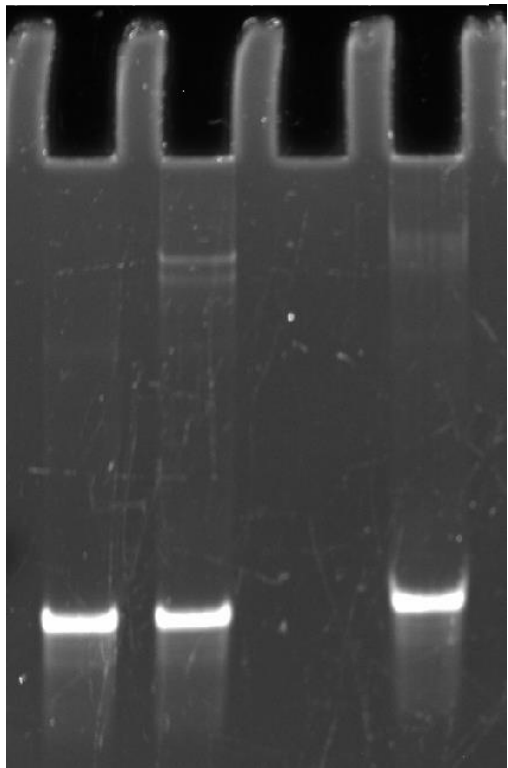

After

|            |   |   |   |   |
|------------|---|---|---|---|
| DNA        | + | + | - | + |
| BATF3/JUNB | - | + | - | + |
| IRF8       | - | - | - | + |

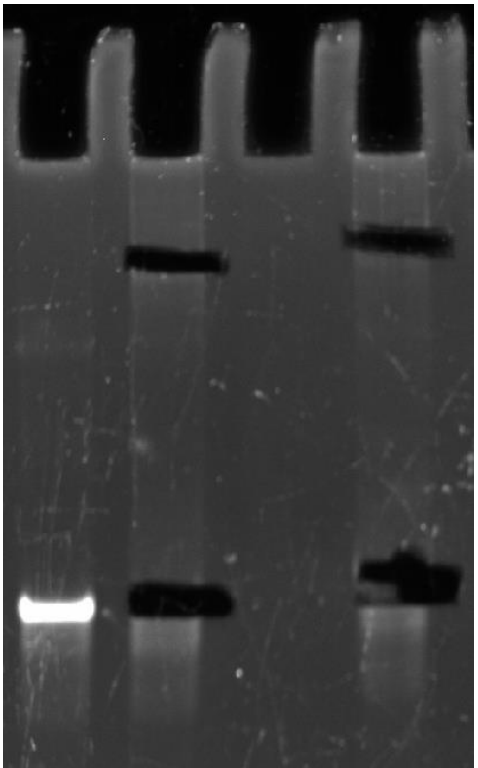

Randomized target

Supplement: Supplementary file 1 — Additional file 1. EMSA gel of protein-DNA complexes. An example of an electrophoretic mobility shift assay (EMSA) gel with proteins BATF3, JUNB and IRF8 and randomized DNA libraries BATFx–JUNB Spec-seq Oligo 1–3 (Fig. 1). First lane shows band for unbound DNA. Second lane includes band for BATF3/JUNB complex with DNA (bound band). Fourth lane includes BATF3/JUNB/IRF8 and shows a more diffuse band higher in the gel than the band in lane 2. The gel picture on the right is taken after cutting out the bands and shows the extent of each band that is cut out for DNA extraction and sequencing. [file 12867_2018_106_MOESM1_ESM.pdf]

A

|         |                   |
|---------|-------------------|
| TRE 0sp | <b>TGAG</b> TCAN  |
| TRE 1sp | N <b>TGA</b> GTCA |
| CRE     | <b>TGAC</b> GTCA  |

B

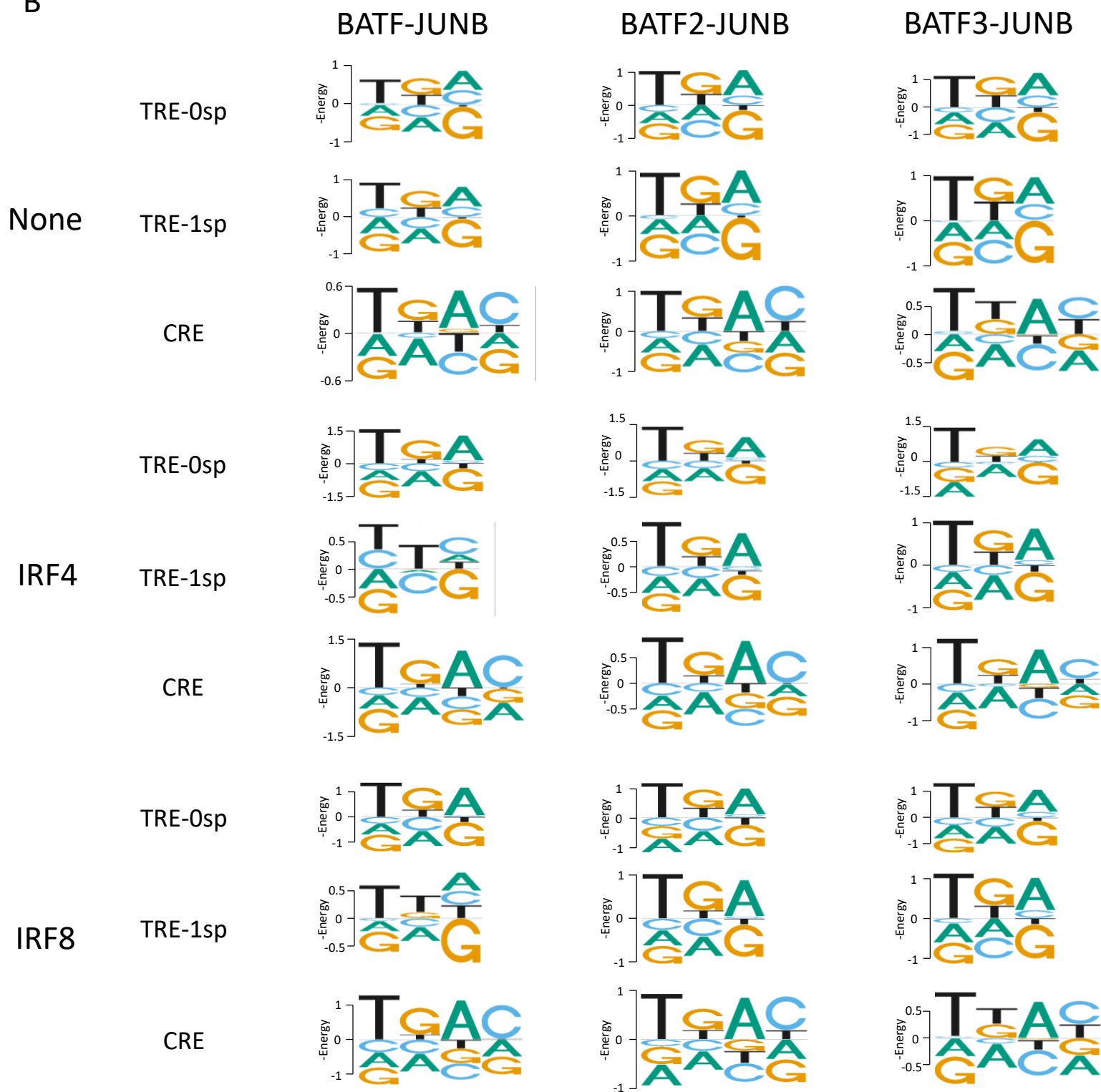

Supplement: Supplementary file 6 — Additional file 6. Half site analysis for BATFx-JUNB-IRFx. (A) Single variants from half sites of these oligos in the library were used to generate energy logos. Bolded positions represent the half sites generated in B. (B) Energy logos from Spec-seq results of BATFx-JUNB-IRFx. The Y-axis is negative energy so the preferred sequence is on the top. [file 12867_2018_106_MOESM6_ESM.pdf]
