# Supplementary material for: Quantitative profiling of BATF family proteins/JUNB/IRF hetero-trimers using Spec-seq
Source: BMC Mol Biol. 2018 Mar 27;19:5. doi: 10.1186/s12867-018-0106-7 (PMC5869772; doi:10.1186/s12867-018-0106-7)
Supplement: Supplementary file 3 — Additional file 3. Energy PWMs from each experiment. For each binding reaction an energy PWM is determined from the consensus sequence and the energy differences for all single variants of the consensus. Each PWM is labeled with the figure of the Logo based on that PWM. [file 12867_2018_106_MOESM3_ESM.docx]

PWMs for logos in figure S1 and S2

Figure S1C. BATF-JUNB TRE

1 2 3 4 5 6 7

A 0.41 0.6 -1.31 0.42 0.3 -0.21 -1.11

C 0.11 0.49 0.08 -0.42 0.93 -0.88 0.58

G 0.58 -0.88 0.93 -0.42 0.08 0.49 0.11

T -1.11 -0.21 0.3 0.42 -1.31 0.6 0.41

Figure S1C. BATF-JUNB CRE

1 2 3 4 5 6 7 8

A 0.46 0.64 -1.14 0.55 -0.15 0.33 -0.34 -1.29

C 0.23 0.37 0.54 -0.87 0.47 0.27 -0.68 0.6

G 0.6 -0.68 0.27 0.47 -0.87 0.54 0.37 0.23

T -1.29 -0.34 0.33 -0.15 0.55 -1.14 0.64 0.46

Figure S1C. BATF2-JUNB TRE

1 2 3 4 5 6 7

A 0.31 0.34 -0.88 0.16 0.27 0 -0.91

C 0.2 0.32 -0.03 -0.16 0.63 -0.66 0.4

G 0.4 -0.66 0.63 -0.16 -0.03 0.32 0.2

T -0.91 0 0.27 0.16 -0.88 0.34 0.31

Figure S1C. BATF2-JUNB CRE

1 2 3 4 5 6 7 8

A 0.39 0.59 -1.1 0.49 -0.11 0.3 -0.32 -1.14

C 0.21 0.36 0.49 -0.93 0.55 0.31 -0.62 0.53

G 0.53 -0.62 0.31 0.55 -0.93 0.49 0.36 0.21

T -1.14 -0.32 0.3 -0.11 0.49 -1.1 0.59 0.39

Figure S1C. BATF3-JUNB TRE

1 2 3 4 5 6 7

A 0.41 0.42 -1.03 0.22 0.31 -0.05 -1.1

C 0.2 0.45 -0.08 -0.22 0.8 -0.82 0.49

G 0.49 -0.82 0.8 -0.22 -0.08 0.45 0.2

T -1.1 -0.05 0.31 0.22 -1.03 0.42 0.41

Figure S1C. BATF3-JUNB CRE

1 2 3 4 5 6 7 8

A 0.35 0.48 -0.74 0.4 -0.14 0.22 -0.26 -0.9

C -0.04 0.17 0.47 -0.53 0.27 0.05 -0.39 0.59

G 0.59 -0.39 0.05 0.27 -0.53 0.47 0.17 -0.04

T -0.9 -0.26 0.22 -0.14 0.4 -0.74 0.48 0.35

Figure S2C. BATF-JUNB-IRF4

1 2 3 4 5 6 7 8

A -0.08 0.1 -0.16 0.18 0.06 -0.49 -0.43 -1.16

C 0.21 0.1 0.13 -0.04 0.43 0.56 0.8 0.74

G 0.12 -0.15 0.05 -0.24 -0.59 -0.16 0.26 0.38

T -0.25 -0.04 -0.01 0.1 0.11 0.09 -0.62 0.05

Figure S2C. BATF-JUNB-IRF8

1 2 3 4 5 6 7 8

A 0.07 -0.09 -0.06 0.13 -0.12 -0.17 -0.16 -0.86

C 0.04 0.32 0.04 -0.14 0.22 0.26 0.42 0.66

G 0.11 -0.1 0.15 -0.01 -0.15 -0.01 0.29 0.25

T -0.22 -0.12 -0.13 0.02 0.06 -0.08 -0.55 -0.05

Figure S2C. BATF2-JUNB-IRF4

1 2 3 4 5 6 7 8

A -0.13 -0.16 -0.48 0.51 0.3 -0.84 -0.81 -1.57

C 0.33 0.11 0.37 0.15 0.62 0.84 1.25 0.83

G 0.19 0.24 0.29 -0.57 -0.91 -0.16 0.23 0.71

T -0.39 -0.19 -0.18 -0.09 -0.02 0.16 -0.67 0.03

Figure S2C. BATF2-JUNB-IRF8

1 2 3 4 5 6 7 8

A 0.05 -0.05 -0.18 0.14 0.06 -0.29 -0.27 -1.13

C 0.1 0.24 0.27 -0.36 0.28 0.32 0.65 0.42

G 0.17 -0.34 0.21 -0.06 -0.32 0 0.17 0.46

T -0.31 0.15 -0.31 0.29 -0.02 -0.03 -0.54 0.25

Figure S2C. BATF3-JUNB-IRF4

1 2 3 4 5 6 7 8

A -0.2 -0.16 -0.27 0.46 0.35 -0.64 -0.63 -1.22

C 0.31 0.18 0.32 0.15 0.4 0.61 1.02 0.65

G 0.22 0.08 0.19 -0.6 -0.71 -0.12 0.15 0.52

T -0.34 -0.09 -0.23 -0.01 -0.04 0.16 -0.54 0.05

Figure S2C. BATF3-JUNB-IRF8

1 2 3 4 5 6 7 8

A -0.04 -0.02 -0.28 0.29 0.02 -0.1 -0.05 -0.59

C 0.1 0.2 0.24 0 0.15 0.2 0.32 0.34

G 0.18 -0.16 0.23 -0.33 -0.14 -0.03 0.07 0.23

T -0.23 -0.01 -0.19 0.04 -0.03 -0.07 -0.33 0.02
